# 1880. Comparative Analysis Between Elderly And Non-Elderly Tuberculosis Patients

**DOI:** 10.1093/ofid/ofad500.1708

**Published:** 2023-11-27

**Authors:** Fatma Hammami, Makram Koubaa, Khaoula Rekik, Amal Chakroun, Fatma Smaoui, Chakib Marrakchi, Mounir Ben Jemaa

**Affiliations:** Infectious Diseases Department, Hedi Chaker University Hospital, University of Sfax, Tunisia, Sfax, Sfax, Tunisia; Infectious Diseases Department, Hedi Chaker University Hospital, University of Sfax, Tunisia, Sfax, Sfax, Tunisia; Infectious Diseases Department, Hedi Chaker University Hospital, University of Sfax, Tunisia, Sfax, Sfax, Tunisia; Infectious Diseases Department, Hedi Chaker University Hospital, University of Sfax, Tunisia, Sfax, Sfax, Tunisia; Infectious Diseases Department, Hedi Chaker University Hospital, University of Sfax, Tunisia, Sfax, Sfax, Tunisia; Infectious Diseases Department, Hedi Chaker University Hospital, University of Sfax, Tunisia, Sfax, Sfax, Tunisia; Infectious Diseases Department, Hedi Chaker University Hospital, University of Sfax, Tunisia, Sfax, Sfax, Tunisia

## Abstract

**Background:**

Tuberculosis (TB) remain a public health issue worldwide. All ages might be involved. With the rise of the elderly population, which is more susceptible to TB disease, an increase of TB among this age group is reported. We aimed to compare clinical features and treatment outcomes between elderly and non-elderly TB patients.

**Methods:**

We conducted a retrospective study including all patients hospitalized in the infectious disease department for extrapulmonary TB between 1990 and 2022. Patients aged ≥ 60 years were considered as elderly TB patients.

**Results:**

We encountered 488 non-elderly patients (79%) and 130 elderly patients (21%). Previous medical history of personnel TB (13.8% vs 6.6% ; p=0.007), cancer (10.8% vs 3.7% ; p=0.001) and diabetes mellitus (13.8% vs 3.3% ; p< 0.001) were significantly more frequent among the elderly group. Fever (55.4% vs 54.1% ; p=0.794), loss of appetite (49.2% vs 43.1% ; p =0.213), weight loss (38.5% vs 41% ; p=0.603) and night sweats (33.8% vs 30.8% ; p=0.506) were the revaling symptoms among elderly and non-elderly group. Osteoarticular TB (24.6% vs 11.3% ; p< 0.001) and urogenital TB (20% vs 8.4% ; p< 0.001) were significantly more frequent among the elderly group. Lymph node TB was significantly less frequent among the elderly group (37.7% vs 50.9% ; p=0.007). The elderly patients received separate tablets of antitubercular therapy more frequently (63.1% vs 49.8% ; p=0.007). Side effects of treatment were reported among the elderly (30.8%) and the non-elderly (34.6%), with no significant difference (p=0.408). Complications (22.3% vs 19.3% ; p=0.440), sequelae (13.8% vs 11.9% ; p=0.545), relapse (1.5% vs 4.5% ; p =0.119) and death (2.3% vs 1.8% ; p=0.723) were noted among elderly and non-elderly group, with no significant difference.

**Conclusion:**

Although the elderly had more comorbidities, the revealing symptoms and the disease evolution of extrapulmonary TB were similar in comparaison with the non-elderly patients. Osteoarticular and urogenital TB were more frequent among the elderly. Strategies to maintain a close follow-up and adequate social support are maindatory in order to garantee a favorable evolution of TB.

**Disclosures:**

**All Authors**: No reported disclosures

